# Establishment of Constitutive Model and Analysis of Dynamic Recrystallization Kinetics of Mg-Bi-Ca Alloy during Hot Deformation

**DOI:** 10.3390/ma15227986

**Published:** 2022-11-11

**Authors:** Qinghang Wang, Li Wang, Haowei Zhai, Yang Chen, Shuai Chen

**Affiliations:** 1 School of Mechanical Engineering, Yangzhou University, Yangzhou 225127, China; 2 School of Materials Science and Engineering, Hebei University of Technology, Tianjin 300130, China; 3 School of Materials and Energy, Yunnan University, Kunming 650599, China

**Keywords:** Mg-Bi-Ca alloy, hot deformation, constitutive model, dynamic recrystallization kinetics, microstructure, mechanical properties

## Abstract

The flow behavior of the solution-treated Mg-3.2Bi-0.8Ca (BX31, wt.%) alloy was systematically investigated during hot compression at different deformation conditions. In the present study, the strain-related Arrhenius constitutive model and dynamic recrystallization (DRX) kinetic model were established, and the results showed that both two models had high predictability for the flow curves and the DRX behavior during hot compression. In addition, the hot processing maps were also made to confirm a suitable hot working range. Under the assistance of a hot processing map, the extrusion parameters were selected as 573 K and 0.5 mm/s. After extrusion, the as-extruded alloy exhibited a smooth surface, a fine DRX structure with weak off-basal texture and good strength–ductility synergy. The newly developed strong and ductile BX31 alloy will be helpful for enriching low-cost, high-performance wrought Mg alloy series for extensive applications in industries.

## 1. Introduction

Magnesium and its alloys, as one of the green materials, have shown wide application prospects in transportation, electronics and military industry due to its excellent characteristics, i.e., low density, high specific strength/stiffness, good vibration-reducing performance, etc. [1]. In recent years, Mg alloys have also made new breakthroughs in the field of hydrogen storage [2,3]. However, some bottleneck problems still exist, such as bad corrosion resistance, poor formability and strength–ductility trade of dilemma, to a large extent restricting commercial applications. In the past decade, some researchers have tried their best to overcome the imbalance of strength and ductility using rare-earth (RE) elements [4,5] and severe plastic deformation techniques [6]. Nevertheless, the high cost and the complex processing vastly limit the applications of Mg alloys. Thus, the development of low RE-containing, even RE-free Mg alloys, is an imminent requirement.

Up to now, some new Mg alloys, such as Mg-Sm- [7,8], Mg-Ca- [9,10], Mg-Al- [11], Mg-Sn- [12], Mg-Mn- [13,14] and Mg-Zn-based [15] alloys, have been fabricated successfully to achieve this goal. In order to further enrich the existing low-cost, high-performance Mg alloy systems, Mg-Bi-based series alloys have been exploited in recent years, and they have great potential in developing RE-free materials with outstanding comprehensive mechanical properties. Al, Zn, Ca, Mn and Sn elements have been attempted to add into Mg-Bi-based alloys to ameliorate their mechanical properties [16,17,18,19,20,21,22,23]. Among them, interestingly, wrought Mg-Bi-Ca series alloys exhibit unique mechanical properties, which are dependent on hot deformation parameters. For instance, Meng et al. [17,18,19] pointed out that Ca addition could trigger the texture change in as-extruded Mg-Bi-Ca alloys. As extrusion temperature or die-exit speed increased, an off-basal texture feature formed, inducing a high tensile ductility (~40%) in Mg-1.3Bi-0.9Ca (wt.%) alloy [17,18]. In contrast, under the conditions of a low extrusion temperature of 280 °C and die-exit speed of 4 mm/s, a strong basal texture occurred in Mg-1.5Bi-0.8Ca (wt.%) alloy leading to a high tensile yield strength (~394 MPa) [19]. In order to obtain the strength–ductility synergic Mg-Bi-Ca series alloys, a two-step deformed Mg-1.3Bi-0.7Ca (wt.%) alloy, with a tensile yield strength of ~351 MPa and elongation-to-failure of ~13.2%, was successfully prepared by extrusion and subsequent caliber rolling [20]. In summary, such a new highly strong and ductile RE-free Mg-Bi-Ca alloy will be helpful for enriching low-cost and high-performance wrought Mg alloy series to achieve extensive applications in industries.

However, until now, the flow behavior, constitutive model, dynamic recrystallization (DRX) kinetic model and hot processing map of Mg-Bi-Ca series alloys during hot deformation are rarely reported systematically. It is important to understand the hot flow behavior of Mg-Bi-Ca series alloys to obtain the desired microstructure and to achieve excellent mechanical properties. The Arrhenius-type constitutive model is commonly used to describe the hot flow behavior, predicting the hot-deformed microstructure and optimizing the hot processing parameters of Mg alloys [24,25]. In Mg alloys, the chemical composition is a key factor in influencing the DRX kinetic. When the added alloying elements are soluble in a substrate, non-basal slip may be activated easily during hot deformation since these elements (i.e., RE, Ca and Li) obviously change the stacking fault energy for non-basal slip reducing the ratio of critical resolved shear stresses, (CRSSs) between non-basal and basal slips [26,27,28]. Within grains, the activation of massive slip systems accelerates the dislocation rearrangement to form low-angle grain boundaries (LAGBs), which provide a precursor for the generation of large-angle grain boundaries (HAGBs). For example, Li et al. [29] pointed out that Ce addition into AZ80 alloy presented a lower activation energy than that in without Ce. In addition to non-basal slip, contraction twinning and/or double twinning also could occur in the early stage of hot deformation in Y-containing Mg-Sn-Zn alloy, as reported by Wang et al. [30]. These twins offer the DRX nucleation sites, also promoting the DRX process. On the other hand, as alloying elements are presented as compounds, two cases exist. In general, large-sized phases (>1 μm) play a role in increasing the DRX nucleation sites, known as particles stimulate nucleation (PSN). While the phase size is below 1 μm, they tend to delay the motion of grain boundaries inhibiting the DRX process. However, on the DRX mechanism of Mg-Bi-Ca series alloys, it is still unknown.

Therefore, in order to investigate the hot deformation behavior of Mg-Bi-Ca series alloys, in this work, taking a new RE-free Mg-Bi-Ca alloy as an example, hot compressive tests were performed at different conditions. The Arrhenius constitutive model and the DRX kinetics of this alloy were established. Based on the data of hot compressive flow curves, the suitable hot processing range was measured. Finally, the extrusion parameter was selected to gain the fine complete DRX structure with weak off-texture and obtain the strength–ductility balance in an as-extruded Mg-Bi-Ca alloy.

## 2. Materials and Methods

Commercial pure Mg ingot (≥99.99%), Mg-10Bi (wt.%) and Mg-25Ca (wt.%) master alloys were melted at 720 °C in an electric resistance furnace (Shanghai Yuzhi Technology Co., Ltd, China) under a protective atmosphere (SF_6_:CO_2_ = 1:99). After a series of sitting and slagging, the melt was poured into a steel mold with Φ 80 (in diameter) × 200 (in height) mm preheated to 350 °C. The chemical composition of the as-cast alloy was detected by an X-ray fluorescence spectrometer (XRF, LAB CENTER XRF-1800) (Shimadzu, Kyoto, Japan), and the real chemical composition is 96.0 wt.% Mg, 3.2 wt.% Bi, and 0.8 wt.% Ca. It can be labeled as BX31. Before hot compression, the solid solution of the as-cast BX31 alloy was treated at 773 K for 24 h. Subsequently, hot compression tests for samples with a size of Φ 8 × 12 mm (diameter × height) were operated on a Gleeble-3500 thermo-mechanical simulator (DSI North America Corp, Marlton, Evesham, United States) at different conditions (temperatures: 573, 623 and 673 K; strain rates: 0.01, 0.1 and 1 s^−1^). In addition, the solution-treated BX31 alloy was also extruded into the bar with Φ 22 mm in diameter at 573 K using an extrusion ratio of 21:1 and a die-exit speed of 0.5 mm/s by XJ-500 horizontal extruder (WuxiYuanchang Machine Manufacture Co., Wuxi, China). The room-temperature (RT) tensile properties of the as-extruded sample with 25 mm in gauge length and Φ 5 mm in gauge diameter were machined from the as-extruded bar along extrusion direction (ED) and measured using CMT6305-300 kN universal tensile testing machine (MTS Systems Co Ltd, Shanghai, China) at a strain rate of 1 × 10^−3^ s^−1^.

The microstructure could be observed by scanning electron microscopy (SEM, Gemini SEM 300) (Carl Zeiss, Oberkochen, Germany) equipped with an energy dispersive spectrometer (EDS) (Carl Zeiss, Oberkochen, Germany) and electron backscattered diffraction (EBSD, JEOL JSM-7800F) device (Japan Electronics Corporation, Tokyo, Japan). The phase constitutions were identified by X-ray diffraction (XRD, Rigaku D/Max 2500) (Bruker AXS, Karlsruhe, Germany). The preparation of EBSD samples was composed of grinding, washing, blow-drying and electro-polishing at 20 V and 0.03 A for 90 s at 298 K using AC2. The scanning step size was set as 0.5 μm. All EBSD data were analyzed using ATEX software v2.01.3 (ATEX, Metz, France).

## 3. Results and Discussion

### 3.1. Microstructure of the Solution-Treated BX31 Alloy

**Figure 1:**
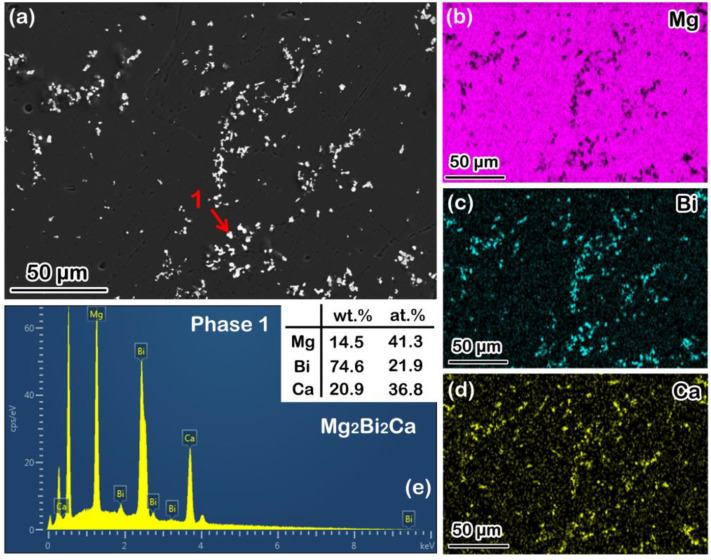
(**a**) SEM image; (**b**–**d**) SEM-EDS mapping scanning results from (**a**); (**e**) SEM-EDS point scanning result from phase 1 marked by red arrow in (**a**).

**Figure 2:**
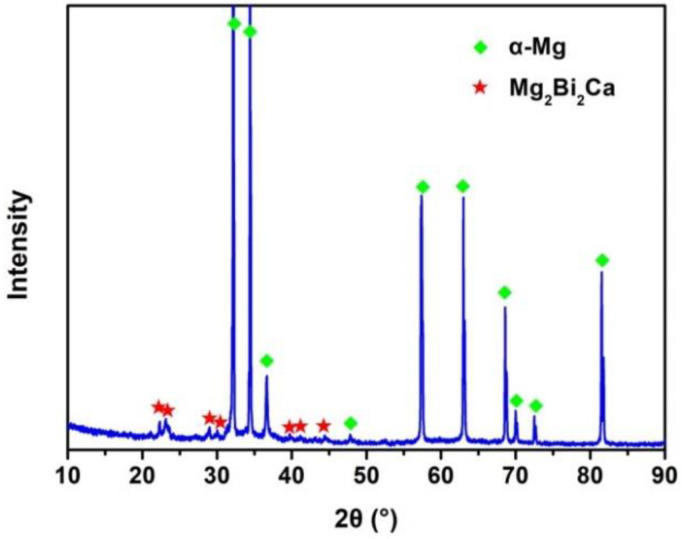
XRD result of the solution-treated BX31 alloy.

Figure 1a shows the SEM image of the solution-treated BX31 alloy with a few bright granular-shaped second phases. The average phase size is about 5 μm. Based on the SEM-EDS mapping scanning results, as shown in Figure 1b–d, these granular-shaped phases mainly consist of Mg, Bi and Ca elements possibly deemed as Mg-Bi-Ca ternary phases. Moreover, by combining the SEM-EDS point scanning result from phase 1 marked by the red arrow in Figure 1a, these ternary phases can be regarded as Mg_2_Bi_2_Ca ones, as displayed in Figure 1e. The reports of Remennik et al. [31], Meng et al. [17,18,19,20] and Liu et al. [32] are consistent with this result. In addition, Mg_3_Bi_2_ phases, even Mg_2_Ca ones, also could be found in wrought Mg-Bi-Ca series alloys [17,18,19,20]. However, the mentioned these two kinds of phases are almost undetectable in the solution-treated BX31 alloy. We argue that these dynamically precipitated fine Mg_3_Bi_2_ and Mg_2_Ca particles are the products of a combined effect from deformation force and temperature. On the other hand, the thermal stability of Mg_2_Bi_2_Ca phases is superior to Mg_3_Bi_2_ and Mg_2_Ca ones, based on the thermodynamics of phase formation [32]. Therefore, in this work, the main phase constitution in the solution-treated BX31 alloy is Mg_2_Bi_2_Ca. In order to further verify this statement, an XRD test was carried out, and the result is shown in Figure 2. We can see that two kinds of characteristic diffraction peaks of phases exist: α-Mg and Mg_2_Bi_2_Ca, which is in accordance with the SEM observation.

### 3.2. Flow Behavior during Hot Compression

**Figure 3:**
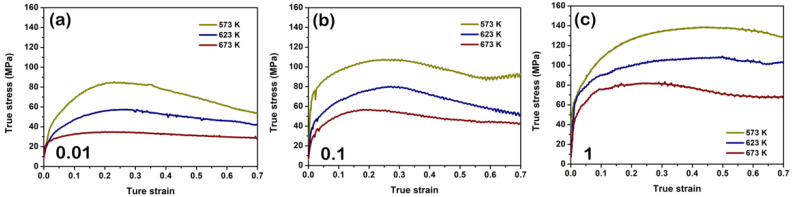
True compressive stress–strain curves at strain rates: (**a**) 0.01 s^−1^; (**b**) 0.1 s^−1^; (**c**) 1 s^−1^.

**Figure 4:**
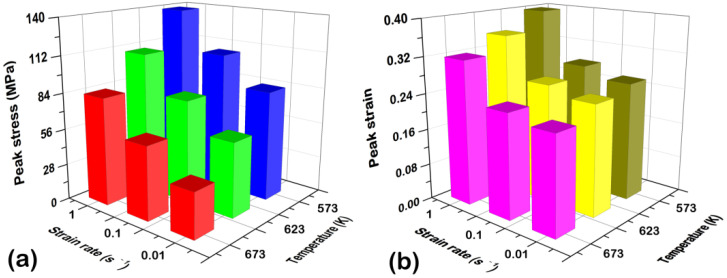
(**a**) Peak stresses and (**b**) peak strains at different deformation conditions. Note that the color difference is to better distinguish the size of the value.

Figure 3 shows the compressive flow curves of the solution-treated BX31 alloy at different deformation conditions. We can see the flow curves contain three stages: (1) Rapid ascent stage. Dislocations accumulate sharply, giving rise to remarkable work hardening; (2) Slow descent stage. The flow stress reaches the peak as the strain increases, and subsequently, dislocations annihilate, giving rise to the decline of the flow stress. This is mainly attributed to the DRX triggering the dynamic softening [24,25,33]; (3) Steady-state flow stage. In this stage, the work hardening and the dynamic softening reach a balance, thereby making the flow stress tend to accord. Figure 4 shows the peak stresses and the corresponding peak strains at different deformation conditions. There exists an obvious tendency for the peak stress increment by decreasing the temperature and increasing the strain rate (see Figure 4a), but the peak strain tends to be decreased (see Figure 4b). This phenomenon is closely associated with the acceleration of the DRX process.

### 3.3. Constitutive Model during Hot Compression

The Arrhenius constitutive models are given [34]:

(1) $$\dot{\varepsilon }=A_{1}\sigma^{n_{1}}exp\left(-\frac{Q}{RT}\right)$$

(2) $$\dot{\varepsilon }=A_{2}exp\left(\beta \sigma \right)exp\left(-\frac{Q}{RT}\right)$$

(3) $$\dot{\varepsilon }=A{\left[sinh\left(\alpha \sigma \right)\right]}^{n}exp\left(-\frac{Q}{RT}\right)$$

where $\dot{\varepsilon }$ is the strain rate; σ is the flow stress; *T* is the temperature; $A_{1}$, $n_{1}$, $A_{2}$, *β*, *A*, *α* and *n* are material constants; *Q* is the activation energy; and *R* is the mole gas constant (8.314 J/mol K). The $n_{1}$, *β* and *α* can be described as follows [35]:

(4) $$\alpha =\frac{\beta }{n_{1}}$$

Zener–Hollomon (*Z*) parameter is introduced to construct the relationship among the *Z*, $\dot{\varepsilon }$ and *T*. It can be shown as follows [36]:

(5) $$Z=\dot{\varepsilon }exp\left(\frac{Q}{RT}\right)$$

For Equation (3) with Equation (5) together, the *σ* as a function of the *Z* is expressed as follows [37]:

(6) $$\sigma =\frac{1}{\alpha }ln\left\{{\left(\frac{Z}{A}\right)}^{\frac{1}{n}}+{\left[{\left(\frac{Z}{A}\right)}^{\frac{2}{n}}+1\right]}^{\frac{1}{2}}\right\}$$

Under the peak stress condition, these material constants of α, *Q*, *n* and *A* are calculated by Equations (7)–(11).

Both sides of Equations (1) and (2) are taken natural logarithms as follows:

(7) $$ln\dot{\varepsilon }=n_{1}ln\sigma +lnA_{1}-\frac{Q}{RT}$$

(8) $$ln\dot{\varepsilon }=\beta \sigma +lnA_{2}-\frac{Q}{RT}$$

**Figure 5:**
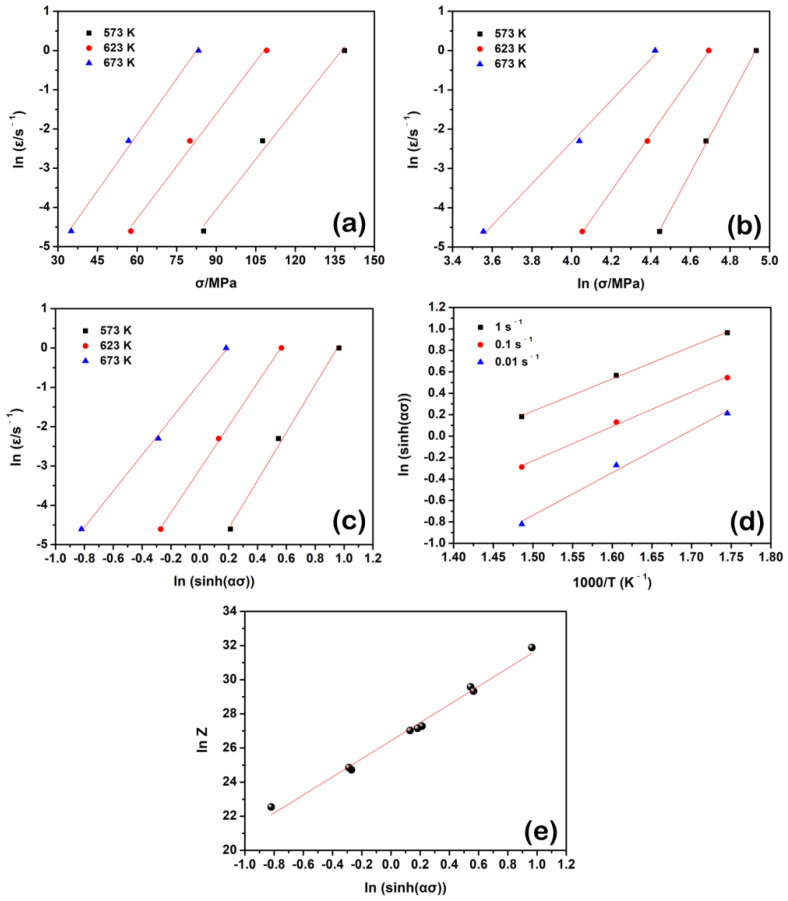
Linear relations of (**a**) $ln\dot{\varepsilon }-ln\sigma_{p}$; (**b**) $ln\dot{\varepsilon }-\sigma_{p}$; (**c**) $ln\dot{\varepsilon }-ln\left[sin\;h\left(\alpha \sigma_{p}\right)\right]$; (**d**) ln[sin h(ασ)]−1/T and (**e**) $lnZ-ln\left[sin\;h\left(\alpha \sigma_{p}\right)\right]$ under the peak stress condition of the solution-treated BX31 alloy.

Figure 5a,b show the linear relationships of $ln\dot{\varepsilon \;}$− lnσ and $ln\dot{\varepsilon \;}$− σ, respectively. The slopes of these two functions are considered the average $n_{1}$ and β values (~7.317 and ~0.089, respectively). According to Equation (4), the α is estimated by ~0.012.

Both sides of Equation (3) are taken natural logarithms as follows:

(9) $$ln\dot{\varepsilon }=nln\left[sin\;h\left(\alpha \sigma \right)\right]+lnA-\frac{Q}{RT}$$

Figure 5c plots the functional relation between $ln\dot{\varepsilon }$ and ln[sinh(ασ)], and the slope can be regarded as the average *n* value (~5.390).

Both sides of Equation (9) are taken partial derivatives as follows:

(10) $$Q=R(\frac{\partial ln\dot{\varepsilon }}{\partial ln\left[sin\;h\left(\alpha \sigma \right)\right]}|T)\left(\frac{\partial ln\left[\sin\;h\left(\alpha \sigma \right)\right]}{\partial \left(1/T\right)}|\dot{\varepsilon }\right)=RnS$$

Figure 5d shows the functional relation of ln[sin h(ασ)]−1/T, and the average *S* value is the slope of this linear relationship, ~3.390. Therefore, the *Q* value at the peak stress condition is about 151.9 kJ/mol, slightly larger than that of pure Mg (~135 kJ/mol [38]). Fine precipitates could effectively inhibit dislocation movement during deformation, which led to an increase in the activation energy during deformation [39]. According to the reports from the literature [17,18,19,20], fine Mg_3_Bi_2_ and Mg_2_Ca particles may be dynamically precipitated from the Mg matrix in Mg-Bi-Ca series alloys, and a part of them was found to distribute at grain boundaries. Such a grain boundary pinning effect further enhances the resistance of grain boundary motion, giving rise to the increment of the activation energy during hot compression. In addition, recently, the *Q* value has been successfully related to the atomistic mechanisms for different engineering materials. Savaedi et al. [40] pointed out that the average *Q* value was close to the weighted self-diffusion activation energy for each element in CoCrFeMnNi alloy. Relevant research was also presented in the report of Jeong et al. [41]. This kind of analysis, considering the atomistic mechanisms, needs more attention in future work. 

Combining Equation (3) with Equation (5), the following relationship is shown:

(11) lnZ=nln[sin h(ασ)]+lnA

The values of *Z* can be calculated at different deformation conditions by taking into the *Q* value. Figure 5e matches the linear function of lnZ − ln[sin h(ασ)], and the *lnA* is ~26.430 as the intercept of this function. As mentioned above, the Arrhenius constitutive model at the peak stress condition can be established as follows:

(12) $$\dot{\varepsilon }=3.008\times 10^{11}{\left[sin\;h\left(0.012\sigma \right)\right]}^{5.390}exp\left(-\frac{1.827\times 10^{4}}{T}\right)$$

**Figure 6:**
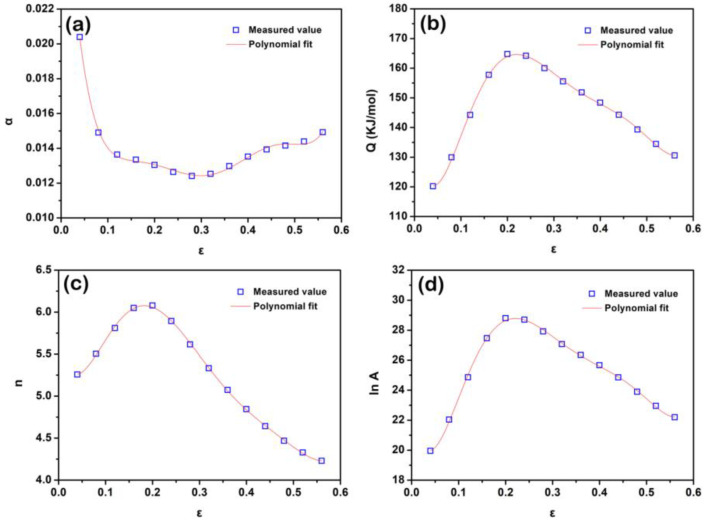
(**a**) *α*, (**b**) *Q*, (**c**) *n* and (**d**) *lnA* as functions of the true strain *ε*.

**Figure 7:**
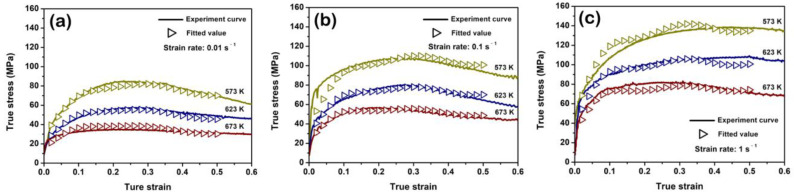
Comparison of experiment flow curves and predicted results of the strain-related Arrhenius constitutive model at different strain rates: (**a**) 0.01 s^−1^; (**b**) 0.1 s^−1^; (**c**) 1 s^−1^.

**Figure 8:**
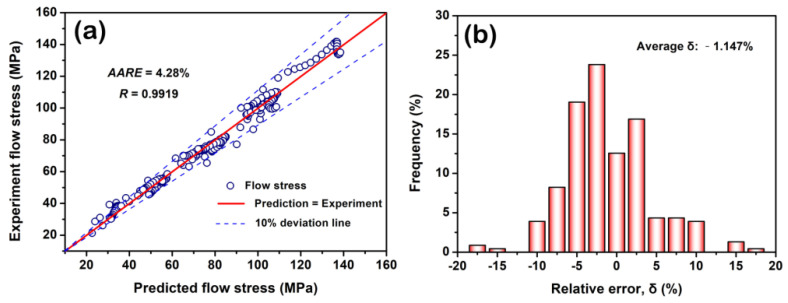
Correlation (**a**) and the relative error (**b**) between experiment and predicted flow stresses by the strain-related Arrhenius constitutive model.

Under the case of the strain-related Arrhenius constitutive model, the material constants of α, *Q*, *n* and *A* are closely associated with the strain. By using 6-order polynomial equations expresses their relations as follows:

(13) $$\alpha \left(\varepsilon \right)=\alpha_{6}\varepsilon^{6}+\alpha_{5}\varepsilon^{5}+\alpha_{4}\varepsilon^{4}+\alpha_{3}\varepsilon^{3}+\alpha_{2}\varepsilon^{2}+\alpha_{1}\varepsilon +\alpha_{0}$$

(14) $$Q\left(\varepsilon \right)=Q_{6}\varepsilon^{6}+Q_{5}\varepsilon^{5}+Q_{4}\varepsilon^{4}+Q_{3}\varepsilon^{3}+Q_{2}\varepsilon^{2}+Q_{1}\varepsilon +Q_{0}$$

(15) $$n\left(\varepsilon \right)=n_{6}\varepsilon^{6}+n_{5}\varepsilon^{5}+n_{4}\varepsilon^{4}+n_{3}\varepsilon^{3}+n_{2}\varepsilon^{2}+n_{1}\varepsilon +n_{0}$$

(16) $$A\left(\varepsilon \right)=exp\left[lnA\left(\varepsilon \right)\right]=exp(A_{6}\varepsilon^{6}+A_{5}\varepsilon^{5}+A_{4}\varepsilon^{4}+A_{3}\varepsilon^{3}+A_{2}\varepsilon^{2}+A_{1}\varepsilon +A_{0})$$

where ε is the strain. The coefficients $\alpha_{0}-\alpha_{6},$ $Q_{0}-Q_{6},$ $n_{0}-n_{6}$ and $lnA_{0}-lnA_{6}$ are listed in Table 1. Figure 6 shows these material parameters as functions of the strain. Based on the strain-related Arrhenius constitutive model, the predicted flow stress values at different strains for each deformation condition can be calculated, and Figure 7 compares the experiment flow stresses and the predicted values at different deformation conditions. It can be seen that the strain-related Arrhenius constitutive model is suitable for the flow curves of the solution-treated BX31 alloy during hot compression. The predictability of this model can be evaluated by the correlation coefficient (*R*) and the average absolute relative error (*AARE*), and the relative error (*δ*) as follows [37,42]:

(17) $$R=\frac{\sum_{i=1}^{n}\left(E_{I}-\bar{E}\right)\left(P_{i}-\bar{P}\right)}{\sqrt{\sum_{i=1}^{n}{\left(E_{i}-\bar{E}\right)}^{2}\sum_{i=1}^{n}{\left(P_{i}-\bar{P}\right)}^{2}}}$$

(18) $$AARE\left(\%\right)=\frac{1}{n}\overset{n}{\underset{i=1}{\sum }}\left|\frac{E_{i}-P_{i}}{E_{i}}\right|\times 100\%$$

(19) $$\delta \left(\%\right)=\left(\frac{E_{i}-P_{i}}{E_{i}}\right)\times 100\%$$

where *n* is the size of flow stress set. $E_{i}$ and $P_{i}$ are the values of experiment flow stress and predicted one, respectively. $\bar{E}$ and $\bar{P}$ are the mean values of $E_{i}$ and $P_{i}$, respectively. The calculated result shows the *R* is ~0.9919 and the *AARE* is ~4.28%, as shown in Figure 8a. Additionally, the δ is deviated at a low range of ~−1.147%, as shown in Figure 8b. It further suggests that this model works well.

### 3.4. DRX Kinetics during Hot Compression

**Figure 9:**
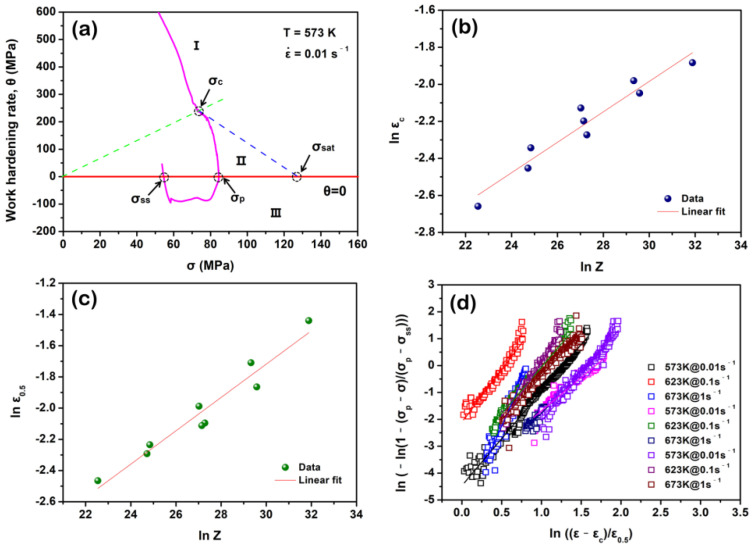
(**a**) *θ*-*σ* curve at 573 K and 0.01 s^−1^. Note that it is divided into three stages marked by I, II and III, respectively; (**b**) relationship between *ln* $\varepsilon_{c}$ and *ln Z*; (**c**) relationship between *ln* $\varepsilon_{0.5}$ and *ln Z*; (**d**) *ln* (*−ln* (($1-\left(\sigma_{p}-\sigma \right)/\left(\sigma_{p}-\sigma_{ss}\right))))$ as a function of *ln* $\left(\left(\varepsilon -\varepsilon_{c}\right)/\varepsilon_{0.5}\right)$.

In order to describe the DRX kinetics model, in general, the following equation can be used and expressed [43,44]:

(20) $$X_{DRX}=1-exp\left[-k_{D}{\left(\frac{\varepsilon -\varepsilon_{c}}{\varepsilon_{0.5}}\right)}^{n_{D}}\right]$$

where $X_{DRX}$ is the DRX area fraction, $\varepsilon_{c}$ is the DRX critical strain, $\varepsilon_{0.5}$ is the strain for the formation of 50% DRX, $k_{D}$ and $n_{D}$ are the material constants. $\varepsilon_{c}$ and $\varepsilon_{0.5}$ can be as a function of the *Z* parameter given as follows [44]:

(21) $$\varepsilon_{c}=B_{1}Z^{m_{1}}$$

(22) $$\varepsilon_{0.5}=B_{2}Z^{m_{2}}$$

where $B_{1}$, $B_{2}$, $m_{1}$ and $m_{2}$ are material constants. The corresponding $\varepsilon_{c}$ and $\varepsilon_{0.5}$ values are obtained by calculating the $\sigma_{c}$ and $\sigma_{0.5}$ ones that represent the stress values, and the corresponding initial DRX and 50% DRX occur, respectively. According to the *θ*-*σ* relationship (*θ* represents the work hardening rate), the $\sigma_{c}$ can be gained. By taking a special case (573 K and 0.01 s^−1^) as an example in Figure 9a, the key flow stresses, i.e., $\sigma_{c}$, peak stress ($\sigma_{p}$), saturated stress ($\sigma_{sat}$) and steady stress ($\sigma_{ss}$), can be measured. In addition, the $\sigma_{0.5}$ can be calculated as follows [44]:

(23) $$X_{DRX}\left(50\%\right)=\frac{\sigma_{p}-\sigma_{0.5}}{\sigma_{p}-\sigma_{ss}}$$

According to the calculation o *θ-σ* curve and Equation (23), Table 2 lists the $\sigma_{c}$, $\sigma_{0.5}$, $\varepsilon_{c}$ and $\varepsilon_{0.5}$ values at different deformation conditions. Figure 9b,c match the linear functions from $ln\varepsilon_{c}$− lnZ and, $ln\varepsilon_{0.5}$− lnZ respectively. Finally, the $\varepsilon_{c}$ and $\varepsilon_{0.5}$ can be expressed:

(24) $$\varepsilon_{c}=0.0116Z^{0.0823}$$

(25) $$\varepsilon_{0.5}=0.0073Z^{0.1067}$$

Figure 9d shows the linear relationship between $ln\left(-ln\left(1-\frac{\sigma_{p}-\sigma }{\sigma_{p}-\sigma_{ss}}\right)\right)$ and $ln\left(\frac{\varepsilon -\varepsilon_{c}}{\varepsilon_{0.5}}\right)$ at different deformation conditions, and the average $k_{D}$ (~0.015) and $n_{D}$ (~3.781) can be determined by the intercept and slope of this function, respectively. Therefore, the DRX kinetics model of the solution-treated BX31 alloy can be expressed as follows:

(26) $$X_{DRX}=1-exp\left[-0.015{\left(\frac{\varepsilon -0.0116Z^{0.0823}}{0.0073Z^{0.1067}}\right)}^{3.781}\right]$$

**Figure 10:**
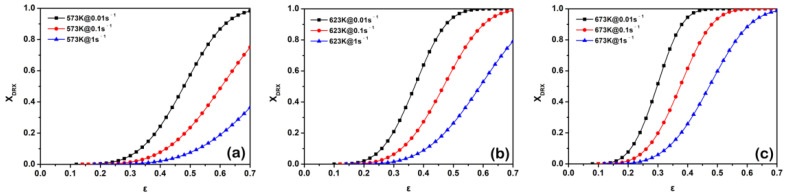
*X*_*DRX*-*ε*_ curves at (**a**) 573 K; (**b**) 623 K; (**c**) 673 K for 0.01, 0.1 and 1 s^−1^.

Figure 10 shows the $X_{DRX}$ as a function of the *ε* at different deformation conditions. As for each curve, as it increased the *ε*, the $X_{DRX}$ gradually increases along an S shape. At the same temperature, the $X_{DRX}$ descends with the strain rate. At a low strain rate of 0.01 s^−1^, the $X_{DRX}$ can reach 100% at each temperature when the *ε* is up to 0.7. As the strain rate enlarges, low temperature obviously declines the DRX degree, especially for the deformation condition of 573 K and 1 s^−1^ (only ~40% at the 0.7 strain).

**Figure 11:**
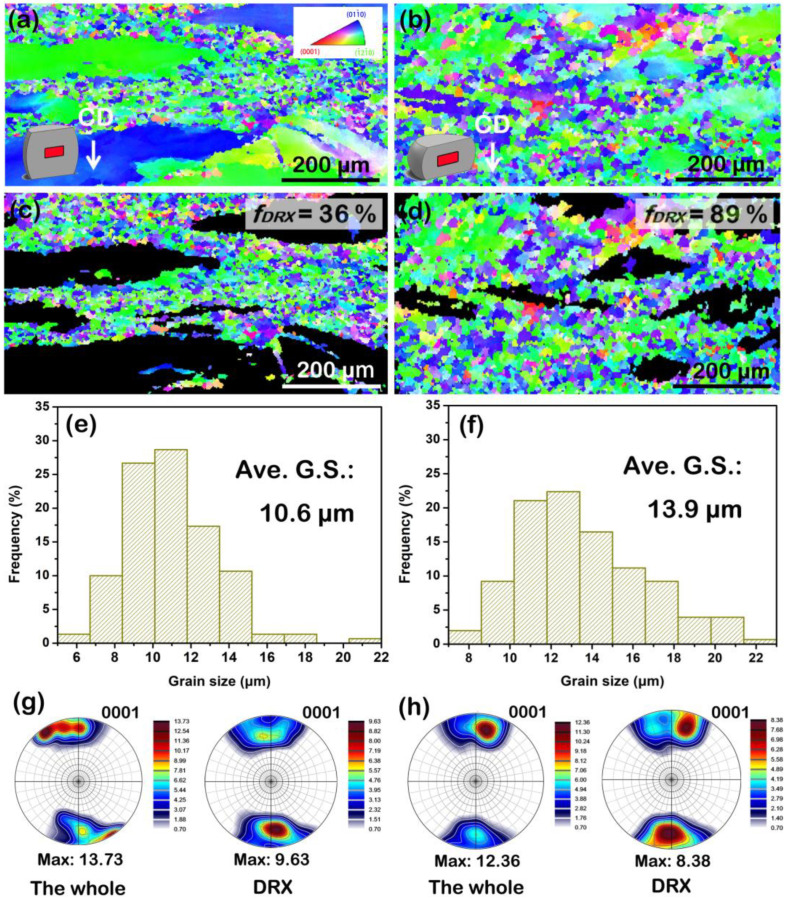
(**a**,**b**) EBSD images of the solution-treated BX31 alloy during the hot compressive strains of 0.3 and 0.5 at 623 K and 0.01 s^−1^, respectively. CD represents the compressive direction; (**c**,**d**) DRX regions selected from (**a**) and (**b**), respectively; (**e**,**f**) grain size distribution maps of DRX regions from (**c**) and (**d**), respectively; (**g**,**h**) (0001) pole figures containing the whole and DRX areas of the samples with 0.3 and 0.5 strains, respectively.

In order to verify the accuracy of the DRX kinetics model, the hot compressive samples at 623 K and 0.01 s^−1^ are selected to observe the DRX area fractions at different strains of 0.3 and 0.5. The result is demonstrated in Figure 11. At 0.3 strain, the DRX and the deformed regions obviously exist (see Figure 11a), and the DRX area fraction is about 36%, as shown in Figure 11c. As the strain increases to 0.5, the DRX region gradually occupies the deformed one (see Figure 11b), and at this moment, the DRX area fraction can reach about 89% shown in Figure 11d. These experiment values are almost accordant with the predicted ones (~30 and ~92%, respectively) using the DRX kinetics model. Therefore, we believe the DRX behavior of the solution-treated BX31 alloy can be forecasted by the DRX kinetics model during hot compression.

Furthermore, we also observed that the DRX nucleation preferentially appears at the original grain boundaries. At a high temperature, the CRSSs of non-basal slips may decline, likely activating the non-basal slips within grains that easily induce the obvious orientation gradient. It is also a type of important DRX mechanism (known as continuous DRX) to promote the formation of the DRXed grains, together with the discontinuous DRX mechanisms (i.e., twinning nucleation, PSN, etc.). As the strain increases, the DRXed grains grow, and its average grain size gradually increases from ~10.6 to 13.9 μm, as seen in Figure 11e,f. Generally, the DRX process plays a key role in refining the grains and weakening the basal texture [43,45]. The DRX area shows a lower basal pole intensity than the whole one in the (0001) pole figures for the 0.3 and 0.5 strains (see Figure 11g,h). Interestingly, the subsequent grain growth induces a weaker basal texture. The related literature has stated that the solute segregation (i.e., Ca) into the boundaries of the basal-oriented grains promoted the preferred growth of the non-basal-oriented ones, which might be a crucial factor in weakening the basal texture [33,46].

### 3.5. Hot Processing Map during Hot Compression

A dynamic material model is valid to describe the hot processing map. In this model, an equation is given [47]:

(27) $$P=G+J=\sigma \dot{\varepsilon }=\int_{0}^{\dot{\varepsilon }}\sigma d\dot{\varepsilon }+\int_{0}^{\sigma }\dot{\varepsilon }d\sigma$$

where *P*, *G* and *J* represent the power dissipated by the workpiece, plastic deformation and microstructural evolution, respectively. The σ as a function of the $\dot{\varepsilon }$ is given [48]:

(28) $$\sigma =K{\dot{\varepsilon }}^{m}$$

where *K* and *m* are a material constant and the strain rate sensitivity coefficient, respectively. Together Equation (27) with Equation (28), *J* is expressed as follows:

(29) $$J=\sigma \dot{\varepsilon }-\int_{0}^{\dot{\varepsilon }}K{\dot{\varepsilon }}^{m}=\frac{m}{m+1}\sigma \dot{\varepsilon }$$

The efficiency of power dissipation (η) is described as follows [35]:

(30) $$\eta =\frac{J}{J_{max}}=\frac{m\sigma \dot{\varepsilon }/\left(m+1\right)}{\sigma \dot{\varepsilon }/2}=\frac{2m}{m+1}$$

Based on the extreme principle, the instability criterion is proposed as follows [27]:

(31) $$\xi \left(\dot{\varepsilon }\right)=\frac{\partial ln\left(\frac{m}{m+1}\right)}{\partial ln\dot{\varepsilon }}+m<0$$

**Figure 12:**
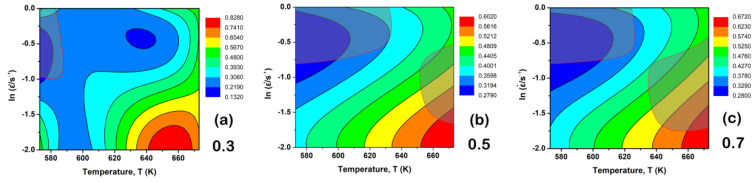
Hot processing maps at different strains: (**a**) 0.3; (**b**) 0.5; (**c**) 0.7.

Figure 12 shows the hot processing maps at the 0.3, 0.5 and 0.7 strains. Note that the shaded regions represent the flow instability ones. At 0.3 strain, the flow instability region locates at 573~590 K and 0.1~1 s^−1^, as shown in the top left corner of Figure 12a. When the strain is up to 0.5, two flow instability regions exist. As for the first region, the temperature and strain rate expand to ~630 K and ~0.5 s^−1^ (shown in the top left corner of Figure 12b), and the new-formed second one is between 650~673 K and 0.2~0.6 s^−1^ (seen in the bottom right corner of Figure 12b). As the strain increases to 0.7, the flow instability area at the top left corner almost never changes, while the second one at the bottom right corner gradually enlarges to 630~673 K and 0.2~0.7 s^−1^ in Figure 12c. Based on the stress–strain curves in Figure 3, the solution-treated BX31 alloy lies in a steady-state flow stage after the 0.7 strain, and thus an appropriate hot processing range can be made up by using the result of Figure 12c.

### 3.6. Analysis of the as-Extruded BX31 Alloy

According to the hot processing range, we can select a suitable extrusion parameter. It has been widely reported that reducing the extrusion temperature or die-exit speed could effectively obtain fine grains to improve the mechanical properties of Mg alloys [15,21,49]. Therefore, 573 K and 0.1 s^−1^ are selected as the deformation parameters within the range for hot processing to perform the extrusion process. The average strain rate ($\bar{\dot{\varepsilon }}$) is a function of die-exit speed ($V_{R}$) at a given extrusion ratio ($E_{R}$), billet diameter ($D_{B}$) and extrudate diameter ($D_{E}$) as follows: [50]:

(32) $$\bar{\dot{\varepsilon }}=\frac{6D_{B}{}^{2}V_{R}lnE_{R}}{D_{B}{}^{3}-D_{E}{}^{3}}$$

In this work, $E_{R}$, $D_{B}$ and $D_{E}$ were set as 21:1, 85 and 22 mm, respectively. Thus, the corresponding extrusion parameters contain the extrusion temperature of 573 K and the die-exit speed of 0.5 mm/s.

**Figure 13:**
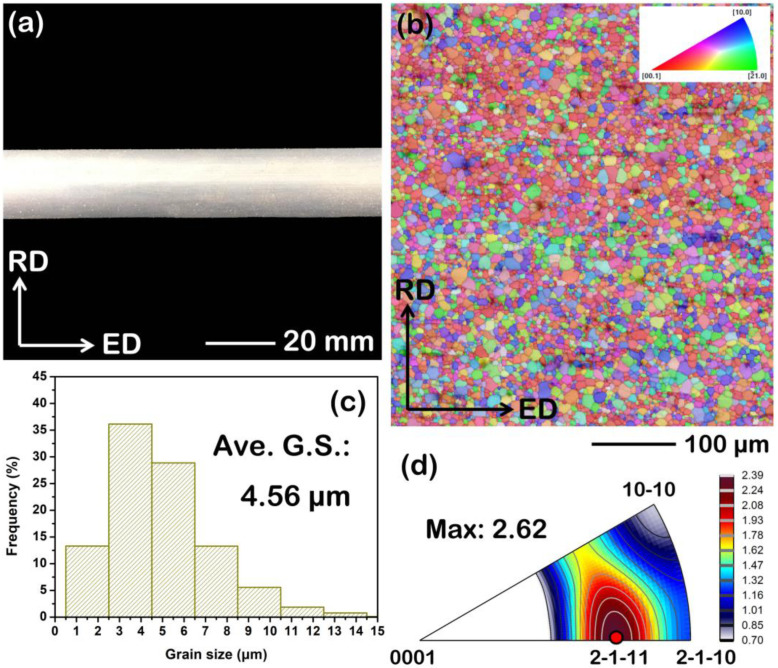
Microstructure analysis of the as-extruded BX31 alloy from longitudinal section (ED-RD plane, RD represents radial direction): (**a**) macroscopic morphology; (**b**) EBSD map; (**c**) grain size distribution map; (**d**) inverse pole figure.

The solution-treated BX31 alloy is perfectly extruded into a bar at a given deformation condition. Figure 13a shows the macroscopic morphology of the as-extruded BX31 alloy. It can be seen that its surface is very smooth by the observation of high magnification. Obviously, the selected extrusion condition is fitted for the forming of the solution-treated BX31 alloy. Furthermore, the grain structure and micro-texture feature of the as-extruded BX31 alloy are measured, as shown in Figure 13b–d. According to the EBSD result from the longitudinal section, the as-extruded BX31 alloy exhibits a relatively homogeneous and complete DRX characteristic, and its average grain size is ~4.56 μm. In terms of micro-texture, a typical RE texture orientation of [2-1-11]//ED is found on the inverse pole figure, accompanied by the maximum pole intensity of ~2.39. Such a texture was also obtained in other wrought Ca-containing Mg alloys [17,18,51,52]. Ca is considered a special element, such as RE ones, being added into Mg alloys to change the stacking fault energies of non-basal slips, thereby to some extent promoting the activation of non-basal dislocations during extrusion. This may be a crucial factor for a typical RE texture formation in the as-extruded BX31 alloy. The detailed DRX mechanism for this alloy during extrusion can be revealed in future work.

**Figure 14:**
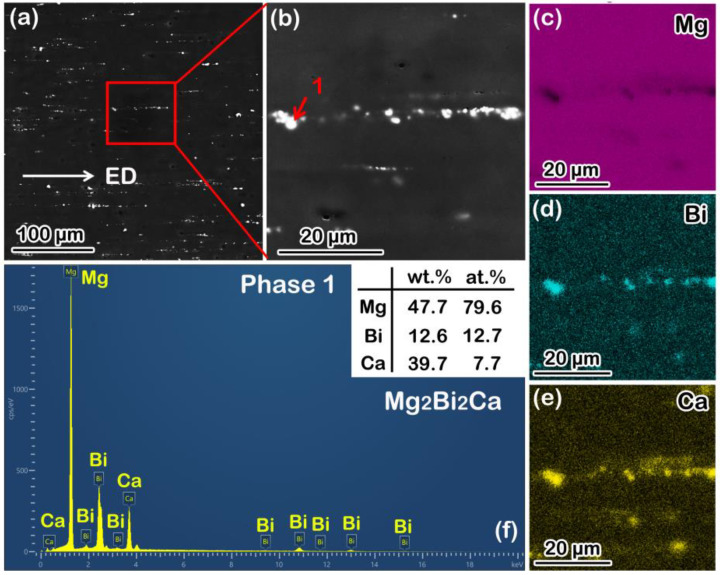
SEM analysis of the as-extruded BX31 alloy from longitudinal section: (**a**) SEM image; (**b**) high magnified view from red frame in (**a**); (**c**–**e**) SEM-EDS mapping scanning results containing Mg, Bi and Ca elements from (**b**); (**f**) SEM-EDS point scanning result from phase 1 marked by red arrow in (**b**).

Additionally, the SEM analysis of the as-extruded BX31 alloy is shown in Figure 14. There exist some granular-shaped phases distributed along ED, and their average sizes are ~2 μm (see Figure 14a,b). Based on the SEM-EDS mapping scanning results shown in Figure 14c–e, these phases mainly also consist of Mg, Bi and Ca elements. Combined with the SEM-EDS point scanning result from phase 1 marked by red arrow in Figure 14b, these phases also can be considered as Mg_2_Bi_2_Ca ternary phases. It indicates that they belong to the broken remains from the second phase in the solution-treated BX31 alloy after extrusion. These fine second phases accelerate the DRX nucleation, known as the PSN effect.

**Figure 15:**
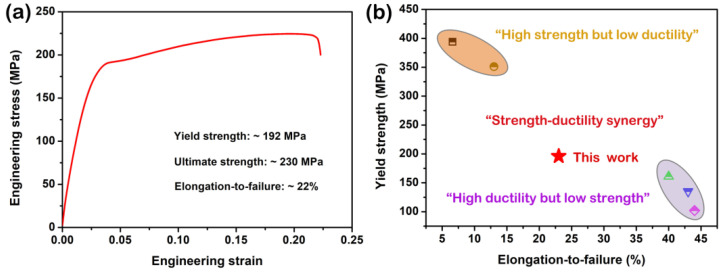
(**a**) Tensile engineering stress–strain curve of the as-extruded BX31 alloy; (**b**) relationship between the ultimate strength and the elongation-to-failure for this work (marked by red star) and the reported other Mg-Bi-Ca series alloys (labeled by different symbols). Note that orange and gray circles represent “high strength but low ductility” and “high ductility but low strength” regions, respectively. Reproduced with permission from Refs. [17,18,19,20]. Copyright 2020, 2022, Elsevier, MDPI.

Figure 15a shows the RT tensile engineering stress–strain curve and the corresponding tensile data of the as-extruded BX31 alloy. It exhibits a relatively high yield strength of ~192 MPa, excellent ultimate strength of ~230 MPa and the desired elongation-to-failure of ~22%. Figure 15b plots the relationship between the yield strength and the elongation-to-failure for this work and the reported other Mg-Bi-Ca series alloys [17,18,19,20]. They are divided into three groups: (1) high strength but low ductility, (2) strength–ductility synergy and (3) high ductility but low strength. Obviously, the reported other Mg-Bi-Ca series alloys have a strength–ductility trade-off dilemma. In comparison, the as-extruded BX31 alloy provides a good balance. Such excellent mechanical performance is mainly attributed to three aspects: (1) Fine grain size. According to the Hall–Petch law [15,21,49], fine grains can offer a considerable number of grain boundaries, effectively hindering the dislocation motions, thereby enhancing the strength of this alloy; (2) Homogeneous complete DRX structure. On the one hand, the high homogeneity of microstructure to some extent reduces the degree of stress concentration, and on the other hand, the low residual dislocation density is beneficial for uniform plastic deformation; (3) The formation of off-basal texture. Generally, weakening basal texture benefits the improvement of the ductility in Mg alloys [17,18,52,53]. In this work, the off-basal orientation of [2-1-11]//ED in the as-extruded BX31 alloy induces a high Schmid factor for basal slip (~0.37 from the calculation of EBSD data) during tension. The activation of more basal slips accommodates the larger strains, which is a key factor in increasing the work-hardening capacity and the ductility of this alloy.

## 4. Conclusions

In this work, the hot deformation behavior of the solution-treated BX31 alloy was systematically studied. The strain-related Arrhenius constitutive model presented a high predictability for the flow curves during hot compression. The flow curves followed the DRX-dominated softening mechanism. The DRX nucleation preferentially appears at the original grain boundaries, and subsequently, non-basal slips might be activated to promote the continuous DRX at a high deformation temperature. For a given deformation condition, the DRX benefited from the texture weakening, and interestingly, with increasing the strain, the DRX growth further reduced the basal texture intensity. This result might be attributed to the preferred growth of non-basal-oriented grains due to the solute segregation. According to the hot processing map, a suitable extrusion parameter could be selected, and the as-extruded BX31 alloy showed a balance of strength and ductility. Such good comprehensive tensile properties were mainly attributed to the following three aspects: (i) fine grain size, (ii) homogeneous complete DRX structure and (iii) the formation of off-basal texture. The newly developed strong and ductile BX31 alloy will be helpful for enriching low-cost, high-performance wrought Mg alloy series for extensive applications in industries.

## Figures and Tables

**Table 1:** Coefficients in the polynomial fitting of *α*, *Q*, *n* and *lnA*.

*α*	*Q*	*n*	*lnA*
*α*_0_ = 0.0343	*Q*_0_ = 138.6302	*n*_0_ = 5.6236	*InA*_0_ = 22.9529
*α*_1_ = −0.5172	*Q*_1_ = −1049.6868	*n*_1_ = −22.5556	*InA*_1_ = −181.8720
*α*_2_ = 5.1075	*Q*_2_ = 18,785.7115	*n*_2_ = 432.5720	*InA*_2_ = 3422.1192
*α*_3_ = −25.5741	*Q*_3_ = −110,944.6683	*n*_3_ = −2667.7302	*InA*_3_ = −20,427.6000
*α*_4_ = 67.i.e60	*Q*_4_ = 303,759.5483	*n*_4_ = 7324.5991	*InA*_4_ = 56,149.0301
*α*_5_ = −88.9583	*Q*_5_ = −398,898.1624	*n*_5_ = −9495.3504	*InA*_5_ = −73,886.5001
*α*_6_ = 46.1098	*Q*_6_ = 203,225.5371	*n*_6_ = 4756.1032	*InA*_6_ = 37,684.4203

**Table 2:** *σ_c_*, *σ*_0.5_, *ε_c_* and *ε*_0.5_ values at different deformation conditions.

	Strain Rate (s^−1^)	Deformation Temperature (K)		
		573	623	673
*σ_c_* (MPa)	0.01	67.5	42.9	31.9
	0.1	93.9	56.4	50.3
	1	125.8	78.4	72.8
*σ*_0.5_ (MPa)	0.01	69.1	47.2	32.0
	0.1	102.8	67.5	51.2
	1	130.7	88.8	74.8
*ε_c_*	0.01	0.103	0.086	0.070
	0.1	0.129	0.119	0.096
	1	0.152	0.138	0.111
*ε*_0.5_	0.01	0.123	0.101	0.085
	0.1	0.155	0.137	0.107
	1	0.237	0.181	0.121

## Data Availability

Data available on request.
